# Prevalence of intestinal parasites in referred individuals to the medical centers of Tonekabon city, Mazandaran province

**Published:** 2016-12

**Authors:** Samira Shahdoust, Maryam Niyyati, Ali Haghighi, Eznoallah Azargashb, Mohammad Reza Khataminejad

**Affiliations:** 1 *Department of Parasitology and Mycology, School of Medicine, Shahid Beheshti University of Medical Sciences, Tehran, Iran*; 2 *Department of Health and Social Medicine, Shahid Beheshti University of Medical Sciences, Tehran, Iran*; 3 *Department of Microbiology, Islamic Azad University, Tonekabon Branch, Tonekabon, Iran*

**Keywords:** Intestinal parasites, Prevalence, Tonekabon

## Abstract

**Aim:**

The aim of the present study was to determine the prevalence of intestinal parasites and their relation with socio-demographic data in referred individuals to the medical centers in Tonekabon, Mazandaran province, 2015.

**Background:**

Due to the climatic and ecological conditions in Mazandaran province, determination of the status of intestinal parasites among referred individuals to the medical centers of Tonekabon city can help researchers and healthcare services to prevent and/or control of parasitic infection in this region.

**Methods:**

This cross sectional study was conducted with randomized sampling in 2015 on 820 stool samples. Stool samples were assessed using direct slide smear with saline and Lugol, formalin-ether concentration, Ziehl-Neelsen and trichrome staining. Polymerase Chain Reaction (PCR) using specific primers was conducted for the samples suspected for *Entamoeba histolytica/E.* *dispar *and *Cryptosporidium *spp. One *Cryptosporidium *positive sample in this study was submitted for sequencing.

**Results:**

A total of 444 (54.1%) and 376 (45.9%) were male and female, respectively. Furthermore, 495 (60.4%) and 325 (39.6%) of participants had lived in the urban and rural areas, respectively. Overall, 222 participants (27.1%) were infected with at least one intestinal parasites. Prevalence of pathogenic protozoa (*Giardia lamblia *and *Cryptosporidium *spp.) and helminthes parasites was calculated as 3.1 and 1.2%, respectively. The most common intestinal parasites in this area were: *Blastocystis *153 (18.7%), *Endolymax nana *44 (5.4%), *Entamoeba coli *40 (4.9%), *Giardia lamblia *25 (3%), *Iodamoeba butschlii *22 (2.7%), *Ascaris *5 (0.6%), *Enterobius*

*vermicularis *4 (0.5%), *Trichostrongylus *1 (0.1%) and *Cryptosporidium *1 (0.1%). By sequencing of the positive *Cryptosporidium *isolate using Gp60 gene, *Cryptosporidium parvum *subtype ΠaA16G2R1 was diagnosed.

**Conclusion:**

Protozoa were more abundant than helminthes and *Giardia lamblia *was the most common protozoan pathogen. In this study, no significant association was found between the prevalence and the variables of socio-demographic data. Adequate knowledge and periodic surveillance of the prevalence of parasites and the socio-demographic variables that affect their frequency is important for effective control of parasitic infections.

## Introduction

Gastrointestinal parasitic infections are a common cause of gastrointestinal disorders, especially in developing countries. The role of parasitic infections in the world and their adverse effects on health, economic and social affairs of human being is very important (1,2). Developed countries and possessor of technology, due to improved hygienic measures, have been able to decrease the rate of parasitic infections. (3,4). However entrance of passengers from the infected regions to those countries, has been led to some outbreaks of infection and parasitic diseases. On the other hand, in retarded and developing countries, control of parasitic diseases is faced with obstacles. The hot and humid weather and climate, poverty, malnutrition, lack of clean water, diversity and density of the population, low levels of hygiene, close contact with infected reservoir animals and the lack of continuous research in the field of parasitic infections, are considered as the most important factors in the occurrence and spread of parasitic diseases (5,6).

In Iran, nutritional, health and cultural conditions of the people have caused some parts of our country become as a major focus of intestinal parasitic infection in the world. On the other hand, control of parasitic infections due to biological, economic and cultural conditions is faced with serious problems (7). Different studies have been performed on the prevalence rate of parasitic infections in different parts of the country showing that the prevalence of intestinal parasitic infection is about 2 to 61% (8). A research in Kermanshah showed the prevalence rate 59.13% of intestinal parasitic infections (9). Another studies stated the prevalence rate 46.9% in Kashan (10), 21% in Fereydon_kenar, Mazandaran (11), 27.7% in Ardabil (12) and 8.4% in Ghaemshahr of Mazandaran (7). These results indicate that the prevalence of intestinal parasitic infection is highly variable in Iran.

Since accurate diagnosis and treatment of patients with parasitic infections are important, parasitology methods for the detection of intestinal parasitic disease is considered as useful and beneficial tools. These methods are still valuable, and are often used as a gold standard for diagnosis in clinical laboratories. However, the main problem of these methods are lack of detection of parasites in the cases with low level of parasitemia. These issues result in deficient reports from laboratories and treatment centers (13,14).

Due to the climatic and ecological conditions in Mazandaran province and Tonekabon city, suitable conditions for the distribution and transmission of parasites as well as Lack of a comprehensive study in this region, the current study was performed in this city.

The aim of this study was to determine and evaluate the prevalence of intestinal parasites and the relationship between socio-demographic variables in referred subjects to the medical centers in Tonekabon.

## Material and Methods

**Sampling**

A cross sectional study was performed from May to November 2015 in Tonekabon city, Mazandaran province. The Tonekabon city has a population of about 203000, living in rural and urban areas. The latitude and longitude of Tonekabon is 36.8154 and 50.8716, respectively. The general area of this city is 2140 Km. The average rainfall in the city is 1100 mm. Stool samples were collected randomly according to comments of statistical consultant for 6 months. Sample size was calculated as follow: the sample size was calculated as 818 according to statistics and referencing the Ranjbar bahadori et al. study (7), which in this study in Ghaemshahr city in Mazandaran province the prevalence was reported to be 8.4%. Overall the sample size rose to number 820. All of the samples were collected from referred individuals to the medical centers of the city. All participants filled an informed consent prior giving stool sample.

 After a macroscopic examination of stool samples in terms of color and consistency, all samples were examined by direct microscopic methods (13,14), formalin ether concentration technique and modified Ziehl-Neelsen staining technique (13,14). For more accurate detection of the suspected amoeba samples, trichrome staining and culture of stool samples in liquefied serum media (HSre+s) was also carried out (16,17,18).

Prepared smears were studied by using an optical microscope with magnification of 100x and 400x and stained samples were studied with magnification of 1000x.

*Cryptosporidium *positive and suspected *Entamoeba histolytica/E. dispar *samples were stored in 70% alcohol until DNA extraction.

**DNA extraction and PCR**

DNA was extracted using the DNG Plus kit. To determine the species and subtype of *Cryptosporidium *isolates, nested PCR reactions were performed using two pairs of primers for GP60 gene as follow 5¢ - ATAGTCTCCGCTGTATTC3¢ and 5¢-GCAGAGGAACCAGCATC3’. Required primers for performing the second PCR reaction were included: 5¢-TCCGCTGTATTCTCAGCC-3’ and 5¢-GAGATATATCTTGGTGCG-3¢ (19). PCR was also done for the suspected *E. histolytica/E.dispar *amoebae using specific primer pairs, as mentioned elsewhere (20). To perform PCR reaction, program of thermocycler for 32 cycles was as followed: Initial denaturing 94˚ for 5 minutes, denaturing 94˚ for 30 seconds, annealing 45˚ 30 seconds, extension 72˚ for 30 seconds and final extension 72˚ for 5 minutes. After completing the above steps, to view the PCR product, gel electrophoresis was performed and 400-500 bp band by UV light were visualized. PCR product was then submitted for sequencing at Pishgam Company.

The result of tests were analyzed by SPSS (version 16) with the chi-square test. A p value <0.05 was considered significant. Frequencies and percentages were recorded as descriptive data.

## Results

In this study, 820 samples were collected randomly from subjects referred to health centers of Tonekabon. All samples were examined macroscopically to identify adult helminthes, blood and stool consistency. In total, 27.1% of the attendees were infected with at least one intestinal parasite. 444 participants (54.1%) were male and 376 subjects (45.9 %) were female (Table 1). Prevalence of pathogenic protozoan and helminthes parasites was calculated as 3.1 and 1.2%, respectively (Table 2). Infection in males was 27.9% and in females was 26.1% which indicates that the infection rate in males is a little higher than females. But x2 test with P value of 0.603 did not show a significant relationship between the two genders. In terms of age, there was not a significant relationship between infection and age (P value: 0.452). The 495 of referred individuals (60.4 %) lived in the urban and 325 (39.6 %) lived in rural areas, of which reported cases in urban areas were 28.3% and in rural areas were 25.2%. However, there was no a significant relationship between the prevalence of intestinal parasites and location of residency. Furthermore, there was no significant relationship between education and intestinal parasites. In terms of bowel symptoms, x2 test did not show significant relationship between the frequency of intestinal parasites and symptoms. Of the referred subjects, 73 (8.9 %) and 25 (3%) were in contact with animals and contaminated untreated water, respectively. Only one case was microscopically and molecularly positive for *Cryptosporidium parvum *and no *E. histolytica/E. dispar *was found based on molecular detection. By sequencing of Gp60 gene, *Cryptosporidium *species and subtype was diagnosed as *C.parvum *(ΠaA16G2R1).

**Table 1:** Frequency and percentage of intestinal parasites based on socio-demographic variables in subjects referred to medical centers of Tonekabon, 2015

**Variables**	**Studied cases Numbers**	**Positive cases Numbers**		**%**
**Gender**				
Male	444	124		27.9
Female	376	98		26.1
**Age (year)**				
Under 6	68	13		19.2
6_12	22	7		31.8
12_18	49	10		20.4
**Education**				
Primary school	64		21	32.8
High school	127		30	23.6
Diploma	272		79	29
Graduate	238		65	27.3
Farmer & rancher	77		23	29.9
Student	100		20	20
Housewife	152		42	27.6
Other	231		75	32.5
Contact with contaminated sources				
Yes	98		32	32.7

## Discussion

Parasitic diseases are still a major public health problem in both developing and developed countries. There are many intestinal parasites which could lead to human infection and these parasites can develop a wide variety of clinical symptoms which depends on immunologic, physiologic and demographic factors. Several factors such as overcrowding, weather conditions, lack or absence of health facilities, poverty and in some cases special political situations and regional conflicts are factors affecting the spread of parasitic diseases in these regions of the world. In some cases, developed countries are not safe from the destructive impacts of parasites and many outbreaks have been reported (21,22). Mazandaran province has also given the proper weather conditions, high humidity and rainfall, population density, high water level, extensive agricultural activity, the large number of tourists as well as abandoned animals surrounding humans, to develop a parasites life cycle. A study conducted by Rezaeian and Hooshyar in 1991 revealed a high prevalence (76.4%) of intestinal parasites in rural areas of Tonekabon. In this study *Blastocystis *(31.7%) was the most isolated protozoa and among intestinal helminthes *Trichocephala *(22.5%) and *Ascaris *(16.3%) were reported as the highest reported helminthic parasites (15). Our findings showed that almost all intestinal parasites have a significant decrease. These results may be related to increase of health status and improved sanitation.

In 2009, rate of contamination with intestinal parasites in patients referred to central areas of Mazandaran province was reported to be 17.9% within a year (4). Also, in a study conducted in the western part of Mazandaran province in 2016, the prevalence rate was reported to be 15% (23).

**Table 2:** The prevalence of protozoa and helminthes parasites in subjects referred to medical centers of Tonekabon city, 2015

**Protozoa**	**Frequency**	**Percent (%)**
*Blastocystis*	153	18.7
*Endolimax nana*	44	5.4
*Entamoeba coli*	40	4.9
*Giardia lamblia*	25	3
*Iodamoeba butschilli*	22	2.7
*Cryptosporidium*	1	0.1
*Entamoeba histolytica/dispar*	0	0
**Helminthes**		
*Enterobius vermicularis*	4	0.5
*Ascaris lumbricoides*	5	0.6
*Trichostrongylus*	1	0.1

In this study, we did not find any significant correlation between the prevalence of parasitic infections and studied socio-demographic variables. Based on the results, 27.1% of participants were infected with at least one intestinal parasites. Bahadori and colleagues in 2004 in a study, which was conducted in Ghaemshahr city, reported a prevalence rate of 8.4% of intestinal parasites (7). However, *Blastocystis* is not reported in their results and this may lead to lower prevalence of the reported prevalence. According to the results observed in the present study, the prevalence of intestinal protozoan was 25.9%, intestinal helminthes was 0.9% and protozoa and helminthes co-infection was 0.4%. The results indicate that helminthes infection decreased compared with previous studies (6, 24).

The limitation of the present study was the examination of one stool sample per each case. This may lead to a lower prevalence of intestinal parasites in the examined cases. Additionally, in this study, the scotch tape method was not used to identify *Enterobius vermicularis*, thisthis could be due to the lower percentage of cases of *Oxyuris*. As well as those baermann culture method was not used to detect *Strongyloides stercularis*, use of such proprietary diagnostic method can identify the limits of crabs in this research.

In this study, a significant relationship was not observed between prevalence of intestinal parasites and gender in those who referred to medical centers in Tonekabon city. According to the present result the prevalence in males was 27.9% and in women was 26.1 %. Accordingly, in another study conducted by Badparva, et al. regarding the intestinal parasites in Khorramabad in 2014, a significant association between infection and gender was not observed (24). Moreover, Kiani et al. study in 2014 on patients with gastrointestinal disorder in Nahavand city, stated that the prevalence in men was 33.9% and in women was reported to be 30.4%, which a significant relationship was not observed in this study as well (25). However, through investigation on the intestinal parasites in the residents of rural regions of Mazandaran province, Kia, et al. indicated that the prevalence of these parasites is higher in men than in compared to women (26). With regard to the mentioned studies, it can be stated that the prevalence of intestinal parasites in women and men of different parts of Iran has not same pattern. Although prevalence of intestinal parasites in the males and females in different areas of country has shown some differences, it seems that most of them are not statistically significant.

A significant relationship was not observed between prevalence of intestinal parasites in those who referred to medical centers and age groups. The highest infection relates to a groups of individuals who are in an age range from 6 to 12 years (31.8%) and in the studied jobs, free job with a prevalence of 32.5% has the highest rate of prevalence. After that, the highest prevalence was for groups of farmers and ranchers with 29.9%. The high prevalence rate in parasitic infections in animal husbandry and agriculture can be due to more contact with the soil, water and infected animal.

No significant relationship was observed between the prevalence of intestinal parasites and residency. According to Kiani et al. study also among 618 urban patients, 31.4% and in 683 rural patients, 32.9% were infected with intestinal parasites (25). Overall, inadequate drinking water in some villages, lack of sanitary facilities and contact with soil are possible factors of increased parasitic infections.

No significant relationship was found between education and the prevalence of intestinal parasites. In the elementary group, intestinal parasites (32.8%) were higher than other groups. Higher contamination of intestinal parasites in primary school could be due to the influence of age and failure to comply with personal hygiene. However, a lower number of infected people were in the group with post graduate levels. Also, in other studies similar results were obtained in this field (27-29). Overall*, Giardia lamblia *was the most common protozoan pathogen. Studies across parts of Iran indicated a significant reduction of helminthes infection. This process is due to improved health and living conditions. Adequate knowledge and periodic surveillance of the prevalence of parasites and the demographic variables that affect their frequency is important for effective control of parasitic infections.
